# Mass spectrometric identification of candidate RNA-binding proteins associated with *Transition Nuclear Protein* mRNA in the mouse testis

**DOI:** 10.1038/s41598-019-50052-z

**Published:** 2019-09-20

**Authors:** Bart T. Phillips, Jason G. Williams, Dustin T. Atchley, Xiaojiang Xu, Jian-Liang Li, Andrea L. Adams, Katina L. Johnson, Traci M. Tanaka Hall

**Affiliations:** Epigenetics and Stem Cell Biology Laboratory, National Institute of Environmental Health Sciences, National Institutes of Health, Research Triangle Park, NC 27709 USA

**Keywords:** Proteomics, RNA

## Abstract

Spermatogenesis is a differentiation process that requires dramatic changes to DNA architecture, a process governed in part by Transition Nuclear Proteins 1 and 2 (TNP1 and TNP2). Translation of *Tnp1* and *Tnp*2 mRNAs is temporally disengaged from their transcription. We hypothesized that RNA regulatory proteins associate specifically with *Tnp* mRNAs to control the delayed timing of their translation. To identify potential regulatory proteins, we isolated endogenous mRNA/protein complexes from testis extract and identified by mass spectrometry proteins that associated with one or both *Tnp* transcripts. Five proteins showed strong association with *Tnp* transcripts but had low signal when *Actin* mRNA was isolated. We visualized the expression patterns in testis sections of the five proteins and found that each of the proteins was detected in germ cells at the appropriate stages to regulate *Tnp* RNA expression.

## Introduction

Continual sperm production throughout a male’s lifetime requires tight regulation of mitotic and meiotic divisions, shifts in gene expression and regulatory programs, dramatic morphological transformations, and unique changes to chromatin architecture. About 15% of reproductive-age couples are infertile, with about half of cases attributed to the men^1,2^. Both genetics and environment can negatively impact spermatogenesis, but for many male patients, infertility is idiopathic. Much remains to be discovered about the basic biology of spermatogenesis in order to guide development of better diagnostic and treatment options^3,4^.

Spermatogenesis begins with a spermatogonial stem cell (SSC) that commits to differentiate and then progresses through discrete stages of development until sperm are ultimately produced (reviewed in^5^). Spermatogonia, progenitor germ cells, proceed through meiosis to generate different types of spermatocytes and then undergo morphological transformation from round spermatids to fully differentiated spermatozoa. All cell types along the differentiation process, from the SSC to any of its derivative germ cells, are necessary for normal spermatogenesis. Without any one of those cell types, spermatogenesis would arrest or exhaust itself, either of which would result in infertility.

One dramatic change in germ cells during late stages of spermatogenesis is compaction of DNA in the nucleus. Mouse sperm DNA is ~6-fold more highly condensed than chromosomal DNA. Sperm DNA is not supercoiled nor packaged into nucleosomes, but due to unique germ cell chromatin proteins, the DNA is tightly packaged into the sperm head^6^. Small, basic protamines ultimately replace the standard DNA-associating histones, and Transition Nuclear Proteins (TNPs) are critical factors in the changeover. Until recently it was thought that TNPs displace histones, and then protamines replace TNPs. However, recent data suggest an alternative mechanism by which TNPs help to condense the sperm DNA. A variant histone, H2A.L.2, is incorporated into nucleosomes and initiates the conversion process by opening nucleosomes^7–9^. TNPs then associate with H2A.L.2 and sperm DNA to facilitate subsequent binding and processing of protamines.

Two TNPs are conserved in mammals: TNP1 and TNP2. Both are small, positively-charged proteins. TNP1 is a 6.2-kDa basic peptide, and TNP2 is a 13-kDa protein with N-terminal zinc fingers and a C-terminal basic region. Their activities are similar but not identical. The basic regions bind to and condense naked DNA *in vitro*, but TNP2 has stronger affinity for DNA than TNP1^10^. Both proteins are abundant in spermatids, but TNP1 is present at ~2-fold higher levels than TNP2 in mice. Despite their differences, TNP1 and TNP2 can compensate for one another. Mouse knockout models for each single gene display reduced fertility and abnormal chromatin condensation^11,12^, but male mice lacking expression of both genes are completely infertile^13^. TNPs are not required for histone displacement and protamine deposition, but chromatin condensation, DNA break repair, and protamine P2 processing are defective in double knockout mice, indicating important roles for TNPs in these processes.

TNPs are expressed in a short developmental window in spermatids as chromatin is condensing^14^. This change in the chromatin structure makes it increasingly inaccessible to transcriptional machinery, and as a result, differentiated germ cells cease making new mRNAs^7,14,15^. Thus, new proteins in late spermatogenesis, including TNPs, must be generated from mRNAs that were transcribed and stored in earlier stages of germ cell development. *Tnp1* mRNA is detected in round spermatids but is not translated until later spermatid stages^14^. *Tnp2* mRNA is also detected earlier in spermatogenesis than TNP2 protein. The timing of expression of TNPs is critical for fertility. Premature translation of *Tnp2* mRNA in round spermatids by expressing a transgene with the 3′UTR of human growth hormone produced infertile male mice^16^.

The general uncoupling of transcription and translation at the end of spermatogenesis led us to investigate how *Tnp1* and *Tnp2* translation is regulated. We hypothesized that specific proteins associate with *Tnp* mRNAs to suppress or stimulate their translation. We took a proteomics approach and developed a method to isolate endogenous mRNA species and their associated proteins. We employed biotinylated DNA oligonucleotides and avidin resin to capture endogenous *Tnp* mRNAs and associated molecules from testis extract, and we specifically eluted the complexes with non-biotinylated oligonucleotides. We then used mass spectrometry to identify proteins that were associated with the *Tnp1* and *Tnp2* mRNAs but not *Actin* mRNAs. We hypothesize that the proteins that are specifically enriched by *Tnp1* and *Tnp2* mRNA pulldowns relative to *Actin* mRNA pulldown may function to regulate these transcripts. We assessed the timing of the expression of these potential regulatory proteins in spermatogenesis, their protein expression patterns in mouse testis sections, and their mRNA expression in germ cells by analyses of published single-cell RNA-sequencing (scRNA-seq) data^17^ and bulk RNA-seq data from isolated germ cells^18^. Although we cannot conclude that these are *bona fide* regulators of *Tnp* mRNAs without *in vivo* experiments, we found that the five proteins we identified as associated with *Tnp* mRNAs were expressed in the testis at appropriate times in germ cells, making them viable candidates to regulate *Tnp* mRNA that can be further evaluated by the field.

## Results

### A proof-of-concept experiment to identify proteins associated with an endogenous mRNA

**Figure 1:**
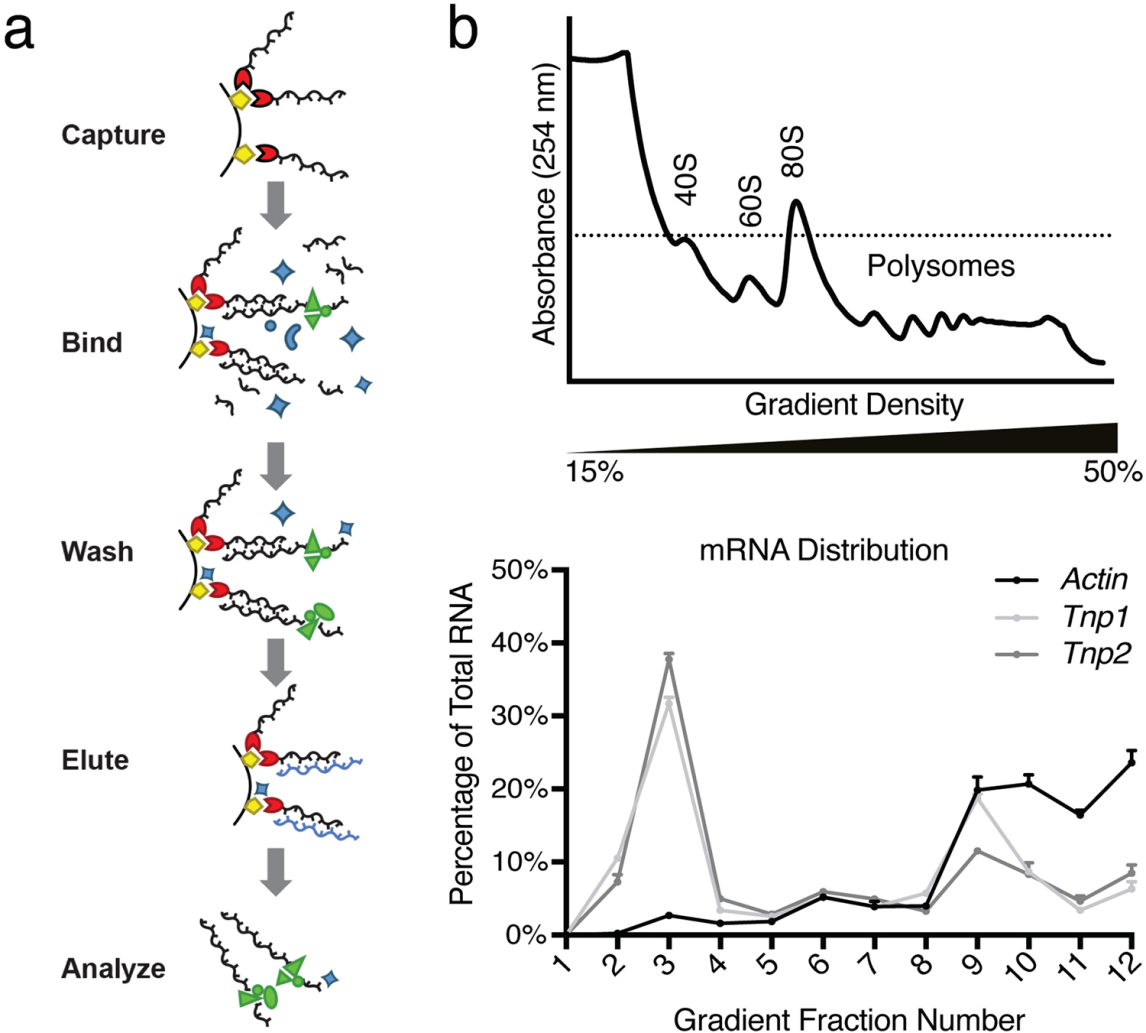
Identification of proteins associated with *Tnp1* and *Tnp2* mRNAs. (**a**) Schematic diagram of *Tnp* mRNA/protein complex isolation. Biotinylated (red) DNA oligonucleotides were captured onto strepatavidin beads (yellow) and then incubated with testis lysate to bind *Tnp* mRNAs with associated proteins (green). After washing to remove unbound mRNAs and proteins, endogenous *Tnp* mRNA/protein complexes were eluted with an excess of complementary oligonucleotide (purple) and subsequently analyzed. (**b**) *Tnp* mRNAs are present in both inactive and actively translated mRNA fractions. A representative polysome profile analysis of adult mouse testis extract is shown (top), detecting the presence of ribosomal subunits (40S and 60S), monosomes (80S), and polysomes by UV absorbance (254 nm). Three biological replicates were performed. *Tnp1*, *Tnp2* and *Actin* mRNAs in sucrose gradient fractions were analyzed using RT-qPCR (bottom). The data are plotted as the percentage of the particular mRNA in a fraction relative to the sum across all gradient fractions. Error bars represent the SEM for the three biological replicates.

We developed and validated a method to isolate endogenous ribonucleoprotein complexes from mouse testis extract and identify the associated proteins (illustrated in Fig. 1a). We designed a biotinylated DNA oligonucleotide complementary to a target mRNA and captured it on avidin resin. We then bound the target mRNA with its associated proteins from testis extract, washed the resin, and then eluted the specifically associated complexes using a competing non-biotinylated oligonucleotide. By using testis extract as the source of the endogenous complexes, we avoided the loss of starting material through isolation of germ cells. In addition, the tissue complexity with germ cells at different stages offered the potential to identify regulatory proteins across spermatogenesis.

As a proof of concept, we tested the ability to isolate *Mapk1* mRNA from adult mouse testis extract and detect associated PUM2, a protein known to bind to the *Mapk1* 3′UTR^19^. We first determined the efficiency of our method to isolate the mRNA of interest. We tested biotinylated DNA oligonucleotides that were complementary to different regions of the target *Mapk1* mRNA (Supplementary Fig. S1a). Using quantitative real-time PCR (RT-qPCR), we measured the amount of target mRNA eluted by competitive oligonucleotide and the amount that was present in the input testis extract. We found that an oligonucleotide targeting the coding region captured 12% of input *Mapk1* mRNA (Supplementary Fig. S1b, Oligo 1). In contrast, oligonucleotides that were complementary to sites in the 5′ or 3′UTR of *Mapk1* mRNA recovered <2% of input target mRNA (Supplementary Fig. S1b, Oligos 2, 3, and 4).

To determine whether PUM2 co-purified with the *Mapk1* mRNA as expected, we selected *Mapk1* mRNA with oligo 1, eluted associated proteins from the avidin resin by adding an excess of the non-biotinylated oligonucleotide, and then analyzed the eluted proteins by western blot (Supplementary Fig. S1c). We detected PUM2 in the elution fraction, confirming its association with the *Mapk1* mRNA. To demonstrate the specificity of this experiment, we found that the RNA-binding protein ELAVL1, which is not expected to bind *Mapk1* mRNA, was not detected in the elution fraction (Supplementary Fig. S1d). As an additional negative control, we also selected *Actin* mRNA using an oligonucleotide complementary to its coding region. We isolated 4% of input *Actin* mRNA, and we did not detect PUM2 protein in this sample (Supplementary Fig. S1e). Collectively, this proof-of-concept experiment confirms the successful isolation of an endogenous ribonucleoprotein complex, making it a suitable method to identify *Tnp1* and *Tnp2* mRNA-associating proteins.

### A proteomics approach to identify proteins associated with Tnp1 and Tnp2 mRNAs

As a first step to identify candidate *Tnp* mRNA regulatory proteins, we used a biochemical approach that would allow us to probe the mouse testis proteome for proteins that could inhibit *Tnp* mRNA translation at earlier stages or stimulate it at later stages. We isolated endogenous *Tnp1* or *Tnp2* ribonucleoprotein complexes from testis lysate using biotinylated oligonucleotides targeting the coding sequences of *Tnp1* or *Tnp2*, which would not interfere with interactions with the 3′UTR that has been shown to be important for regulation^16^. To reduce background from non-specifically associating proteins, we eluted the complexes using non-biotinylated oligonucleotides (Fig. 1a). Using RT-qPCR, we found that we recovered in the eluted fractions ~40% of input *Tnp1* mRNA and ~10% of input *Tnp2* mRNA (Supplementary Fig. S2a). As a control to identify proteins that associate with mRNAs in general or background proteins due to the selection methods, we isolated *Actin* ribonucleoprotein complexes and we recovered ~12% of the input mRNA. Each sample was highly enriched for the selected mRNA versus the other mRNAs (Supplementary Fig. S2b).

We separated the proteins that co-purified with each *Tnp* mRNA by SDS-PAGE and identified proteins from two biological replicates by Nano-scale liquid chromatographic electrospray ionization tandem mass spectrometry (NanoLC-ESI-MS/MS). We also identified proteins from three biological replicates that co-purified with *Actin* mRNA (Supplementary Fig. S2c). To identify candidate *Tnp* mRNA regulatory proteins, we first eliminated from consideration proteins that also co-purified with *Actin* (having a spectral count of >11 in at least one replicate). Since our goal was to identify proteins that regulate specifically *Tnp1* or *Tnp2* mRNA, the housekeeping *Actin* mRNA was used to identify ‘pan’ RNA-binding proteins. This filter also eliminated proteins that might have interacted with the modest but measurable amount of *Actin* mRNA in our *Tnp* mRNA elutions (Supplementary Fig. S2b) and background proteins that were selected non-specifically. Inspection of the remaining list of candidate proteins revealed reasons for confidence in our experimental design. First, the high abundance RNA-binding protein expressed by all germ cells, DDX4 (VASA), was not identified. In addition, RNA-binding proteins with specificity for undifferentiated germ cells (which do not express *Tnp1* or *Tnp2* mRNA), such as PUM2 or RBMY, were not identified in our experiments.

We next scored candidate regulatory proteins using the weighted sums of protein spectral counts in the *Tnp* mRNA samples and identified 69 proteins that had a *Tnp1* + *Tnp2* total weighted sum spectral count >9 (Supplementary Dataset S1). We normalized spectral counts to *Actin* mRNA samples (spectral counts *Tnp*/spectral counts *Actin*) and added the values for two *Tnp1* or *Tnp2* biological replicates and also across all *Tnp1* and *Tnp2* replicates. This list of *Tnp* mRNA-associated proteins contained a large number of splicing factors and other RNA processing factors, including many of the proteins with the highest signal. Surprisingly, these proteins were not detected with *Actin* mRNA. We reasoned that these proteins might be removed upon active translation of the *Actin* mRNA. We analyzed the testis extract by polysome profiling to determine the translational status of *Tnp1* or *Tnp2* vs *Actin* mRNAs and found that *Actin* mRNA is predominantly in the actively translated fractions (Fig. 1b, fractions 9–12) whereas *Tnp* mRNAs are split between inactive (Fig. 1b, fractions 2–4) and active fractions (Fig. 1b, fractions 9–12), which is not surprising as all stages of differentiation are present in the whole testis extract. Although splicing factors and other RNA processing factors may also regulate *Tnp* RNAs^20^, we focused on proteins from other functional categories for further study.

We selected six proteins to examine for potential to regulate *Tnp1* and *Tnp2* expression (Table 1). Five proteins had spectral counts >10 in at least one replicate: Cold shock domain-containing protein E1 (CSDE1) or Upstream of N-Ras (Unr), Embryonic-lethal abnormal visual-like 1 (ELAVL1) or HuR, Insulin-like growth factor 2 mRNA-binding protein 3 (IGF2BP3) or IMP3, Leucine-rich PPR motif-containing protein (LRPPRC) or LRP130, and Matrin-3 (MATR3). Mice null for ELAVL1 are male infertile while none of the others has been studied *in vivo*. We also selected Deleted in Azoospermia-Like (DAZL), because the *Tnp1* and *Tnp2* mRNAs contain putative DAZL binding sites in their 3′UTRs and knockout mice are male infertile.

**Table 1:** Candidate *Tnp1* and *Tnp2* Regulatory Proteins.

Accession #	Gene		Tnp1 Spectral Counts^a^	Tnp2 Spectral Counts^a^
Q91W50	CSDE1	Cold shock domain-containing protein E1	49.1	1.1
P70372	ELAVL1	ELAV-like protein 1	28.9	7.8
Q6PB66	LRPPRC	Leucine-rich PPR motif-containing protein, mitochondrial	20.7	3.4
Q8K310	MATR3	Matrin-3	9.5	9.8
Q9CPN8	IGF2BP3	Insulin-like growth factor 2 mRNA-binding protein 3	11.2	3.9
Q64368	DAZL	Deleted in azoospermia-like	6.4	6.7

^a^Spectral counts reflect the sum of normalized spectral counts over two biological replicate experiments.

### Candidate *Tnp* mRNA regulators are expressed at early developmental stages in the mouse testis

**Figure 2:**
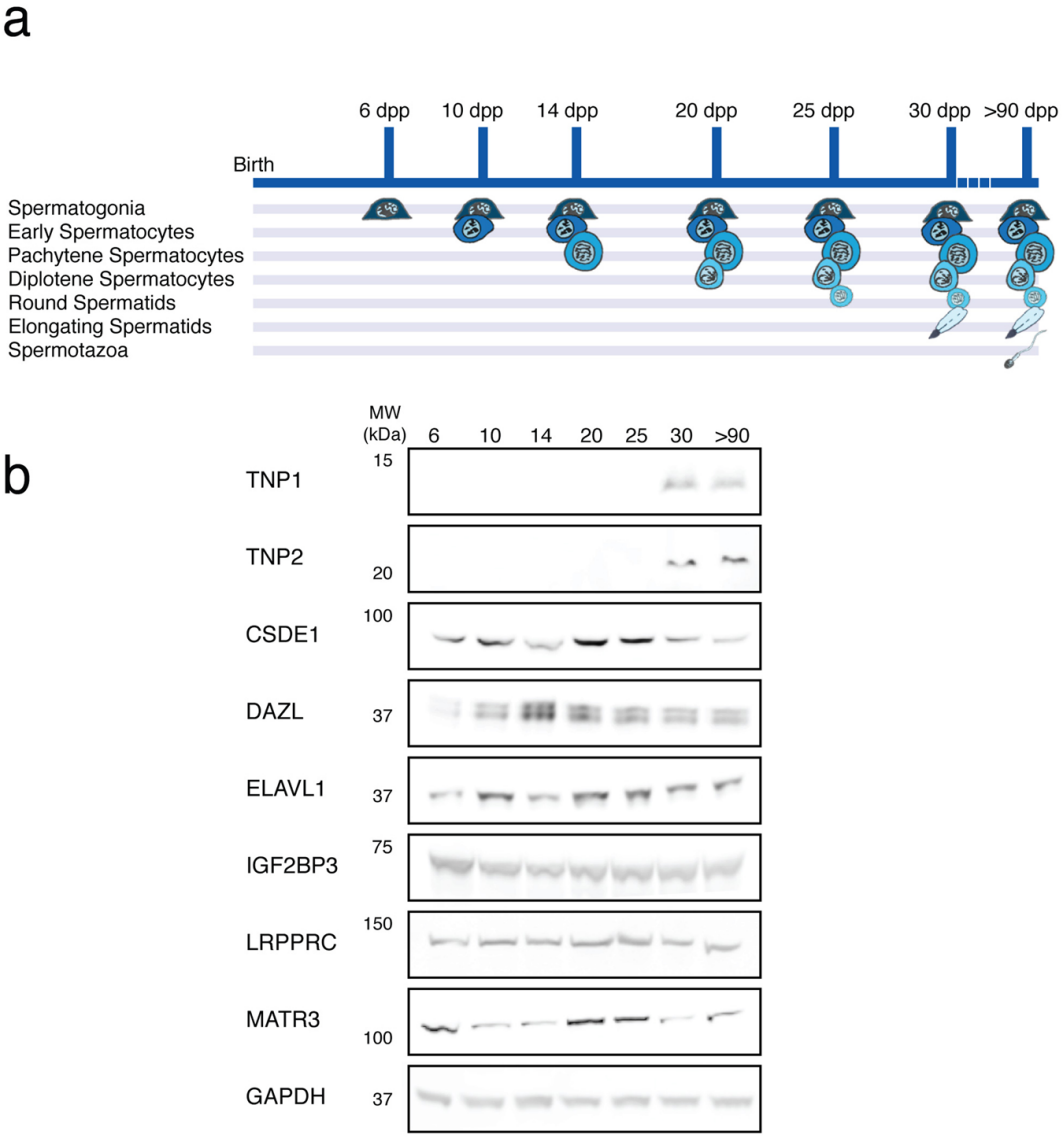
*Tnp* mRNA-associated proteins are expressed early in mouse testis development. (**a**) Diagram of germ cell composition of the mouse testis at different developmental stages. The only germ cells in a testis from a mouse that is 6 dpp are spermatogonia. At 10 dpp, spermatogonia and early spermatocytes in meiosis (preleptotene, leptotene, and zygotene spermatocytes) are present. As development proceeds, pachytene spermatocytes (14 dpp), diplotene spermatocytes (18 dpp), round spermatids (22 dpp), elongating spermatids (30 dpp) and fully differentiated spermatozoa (>90 dpp adult) are included in the testicular repertoire. (**b**) Candidate *Tnp* regulatory proteins are expressed early in spermatogenesis. Relevant bands from representative western blots of mouse testis extracts probed with antibodies recognizing TNP1, TNP2 and the six candidate proteins (CSDE1, DAZL, ELAVL1, IGF2BP3, LRPPRC and MATR3) are shown. Extracts were prepared from mouse testes at seven distinct developmental time points (6, 10, 14, 20, 25, 30 and >90 dpp). Each experiment was performed on three sets of biological replicates. A blot probed for the housekeeping protein GAPDH is shown as a loading reference. Antibodies for TNP1, TNP2, and the six candidate proteins were specific for the respective target proteins by western blot. Full-length blots are presented in Supplementary Fig. S3.

We reasoned that *Tnp* regulatory proteins must be expressed when the mRNAs are expressed, and therefore first assessed the temporal expression of the six candidate proteins across the timeline of spermatogenesis. We took advantage of the developmental nature of the mouse testis, which adds cell types at defined times as mice mature (Fig. 2a). Testes from mice prior to 10 days postpartum (dpp) contain only the most undifferentiated germ cells, spermatogonia. Some of those spermatogonia commit to differentiation, and early spermatocytes are present in the testis along with spermatogonia at 10 dpp. More mature cell types are present at 14 dpp (pachytene spermatocytes), 20 dpp (diplotene spermatocytes), 25 dpp (round spermatids) and 30 dpp (elongating spermatids). In mature mice (>90 dpp), spermatozoa are present in the germ cell repertoire. We could determine the earliest cell types that express TNP1, TNP2, and the candidate regulatory proteins using immunoblots of testis extracts from the different developmental time points. As expected, we found that TNP1 and TNP2 were detected in testis extracts from mice, coincident with the appearance of elongating spermatids. However, the proteins were absent from samples at earlier time points (Fig. 2b), including in the 25 dpp testis that lacks elongating spermatids, but contains round spermatids where *Tnp* mRNAs have been detected^14,21,22^. In contrast, all six candidate *Tnp* mRNA regulatory proteins were detected in testes at the earliest developmental stage (6 dpp) when spermatogonia are the only germ cells present (Fig. 2b and Supplementary Fig. S3). The strength of the western blot signal varied at some time points for CSDE1, DAZL, ELAVL1 and MATR3, but it is difficult to interpret what this means. The extracts were prepared from tissue samples with different cell types expressing target proteins at varying levels. For example, a weaker band intensity for CSDE1 at 14 dpp may indicate that pachytene spermatocytes express lower levels of CSDE1, but the stronger band intensity for the 20 dpp testis extract could be due to higher CSDE1 levels in diplotene spermatocytes or a higher percentage of other cells that express CSDE1 (spermatogonia, early spermatocytes, or non-germ cells) in the 20 dpp testis. We therefore turned to immunohistochemistry to explore which cell types express the candidate *Tnp* regulatory proteins.

### Candidate *Tnp* mRNA regulators are expressed in germ cells that express *Tnp* mRNAs

**Figure 3:**
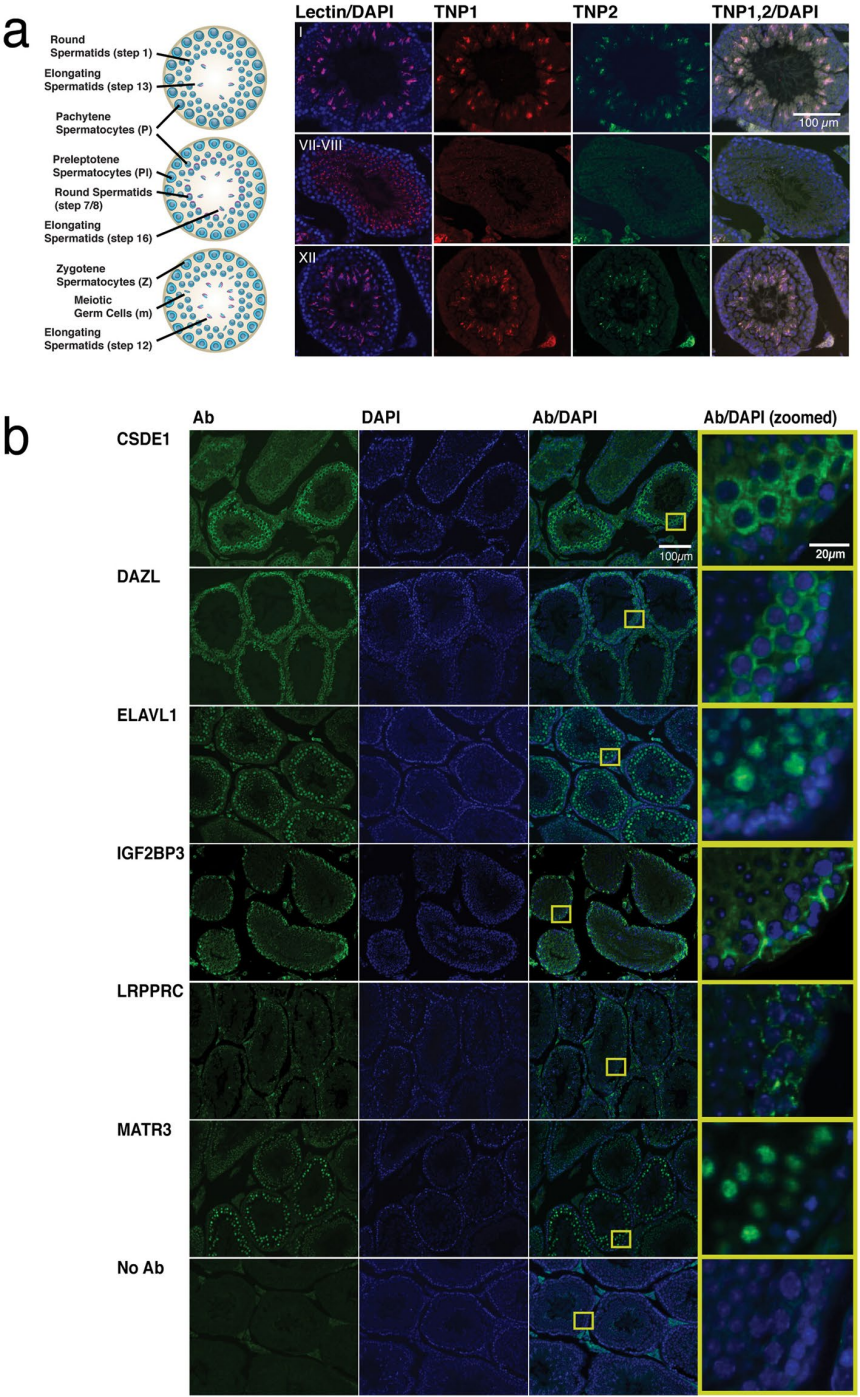
TNP1, TNP2 and candidate RNA-binding proteins are expressed in differentiating germ cells. (**a**) TNP protein expression is restricted to spermatids in specific stages of the mouse seminiferous epithelium. Drawings (left) illustrate the cell types present at stages I, VII–VIII and XII of the spermatogenic cycle (images courtesy of NIEHS). Representative tubule sections from adult mouse testis are shown stained with lectin (magenta) to identify acrosomes for staging of spermatids and DAPI (blue) to identify nuclei. Immunofluorescent detection of TNP1 (red) and TNP2 (green) are shown as individual panels and as a merged image with DAPI staining. (**b**) *Tnp* mRNA-associated proteins are expressed by adult mouse germ cells. Representative adult mouse testis tubule sections with immunofluorescent detection of candidate proteins: CSDE1, DAZL, ELAVL1, IGF2BP3, LRPPRC and MATRIN3. A representative negative control, where primary antibody was omitted from the staining protocol, is also shown. Immunofluorescent detection of candidate proteins (green) and DAPI staining (blue) are shown as individual panels and as a merged image. Zoomed views of the yellow-boxed areas are also shown to the right of the merged images.

We performed immunofluorescent staining of adult mouse testis sections to discover whether the expression patterns of candidate *Tnp* regulatory proteins were consistent with the ability to regulate *Tnp* expression. Mouse spermatogenesis progresses in a synchronized orderly fashion such that waves of developing germ cells are found progressing along the length of tubules^5^. A histological cross-section of a tubule reveals a specific stage of the spermatogenic process (I–XII) that includes characteristic combinations of cell types at particular steps of the spermatogenic cycle (Supplementary Fig. S4). For example, a stage I tubule contains pachytene spermatocytes, step 1 round spermatids, and step 13 spermatids. *In situ* hybridization of adult mouse testis sections has shown that *Tnp1* mRNA is expressed first in step 7 round spermatids (stage VII) and then at higher levels in later stage spermatids (steps 9–13, stages IX-I). *Tnp1* mRNA becomes undetectable by step 14 (stage II–III)^21,22^. TNP1 and TNP2 proteins are expressed exclusively by spermatids near the lumen of the seminiferous epithelium^23,24^. We confirmed this expression pattern by immunofluorescent staining. Lectin visualization of the acrosomes of developing spermatids facilitated tubule staging^25,26^. We detected TNP1 and TNP2 in elongating spermatids in stage I (step 13) and stage XII (step 12) tubules (Fig. 3a, top and bottom panels). Expression decreases in step 15 spermatids (stages IV–VI), and we did not detect TNP1 and TNP2 in step 16 late spermatids (stages VII–VIII)(Fig. 3a, middle panels).

We next examined the expression patterns of the six candidate *Tnp* regulatory proteins in testis sections (Fig. 3b). Below we summarize our observations for each protein. We considered cells with large nuclei closer to the basement membrane to be consistent with spermatocytes and cells with small nuclei and more luminal than the spermatocytes to be consistent with round spermatids.

- CSDE1, which has a known role in translational regulation, was detected in the cytoplasm of germ cells; it was most strongly expressed by cells with large nuclei that appear to be spermatocytes. CSDE1 expression was also detected at a lower level in the cytoplasm of round spermatids with small nuclei that are closer to the lumen than spermatocytes.
- DAZL, a well-known germ cell protein, was also strongly expressed in spermatocytes, but we detected little to no DAZL expression in round spermatids. Comparing the expression patterns of CSDE1 and DAZL illustrates the absence of the more luminal spermatid staining for DAZL.
- Strong nuclear and diffuse cytoplasmic ELAVL1 expression was detected in spermatocytes and spermatids, but was largely absent from cells on the basement membrane and the more mature spermatids that are closest to the lumen. ELAVL1 staining of cells with large nuclei close to the basement membrane is consistent with spermatocytes, and staining of cells with small nuclei that are 3 and 4 layers of germ cells from the basement membrane is consistent with round spermatids.
- IGF2BP3 was detected in the cytoplasm of germ cells. The strongest signal was observed in cells close to the basement membrane, which could be Sertoli cells. Although the signal was weaker, IGF2BP3 was detected in cells with small nuclei that are multiple layers from the basement membrane, consistent with round spermatids. Comparing to the absence of spermatid staining in the DAZL panels is helpful to distinguish the diffuse IGF2BP3 signal in the cells with small nuclei.
- LRPPRC signal was punctate in the cytoplasm. Like IGF2BP3, we detected stronger signal in cells close to the basement membrane, suggesting its expression in spermatogonia and spermatocytes. However, unlike IGF2BP3, LRPPRC was not detected in what we have assigned as putative round spermatids.
- MATR3 is a nuclear protein that had a similar expression pattern to ELAVL1, with no detected signal in cells along the basement membrane; however, the subsequent layers of differentiating germ cells are positive. This indicates the expression of MATR3 in spermatocytes and round spermatids, but little signal is detected beyond that developmental point.

As another method to identify which germ cell types express the candidate proteins, we analyzed published scRNA-seq datasets for mRNA expression and found that the mRNA expression largely coincides with our detected protein expression results. We examined the mRNA expression of candidate genes in the single-cell transcriptome of germ cells from testes of mice at post-natal day 30 or adults at 8 weeks of age^17^. The cell suspension from adult testes had been depleted of sperm by anti-ACRV1-PE or enriched for spermatogonia by anti-CD90.2 (THY1) selection, and both minimally-processed adult cell samples contain excellent representation across cell types. We found that *Csde1*, *Elavl1*, *Igf2bp3*, and *Matr3* mRNAs are expressed in round spermatids as we had detected by immunofluorescent staining of the corresponding proteins (Supplementary Fig. S5a). The signal for *Lrpprc* mRNA is low like the immunofluorescent signal, but consistent with the immunofluorescent staining, the mRNA is detected mainly in spermatogonia and spermatocytes. *Dazl* is strongly expressed in spermatogonia and spermatocytes, but not in spermatids. Examination of published data reporting mRNA expression in isolated germ cell types^18^ also confirms expression of the candidate genes in spermatids where *Tnp1*/*2* are expressed (Supplementary Fig. S5b).

The expression patterns of these mRNAs and proteins indicate that the five candidate proteins we identified as associated with *Tnp* mRNAs appear to be expressed in spermatids where *Tnp* mRNAs are present. LRPPRC may be considered a lower priority candidate due to its relatively poor expression, and DAZL, which we added as a candidate due to the presence of DAZL regulatory elements in the 3′UTR, does not appear to be expressed in the appropriate cells to regulate *Tnp* mRNAs.

## Discussion

The proper regulation of mRNA expression is critical to establishment and maintenance of the germline (reviewed in^27^). mRNA may follow an uninterrupted process of expression, beginning with transcription and processing followed by export from the nucleus for translation. However, biology dictates some instances where mRNAs cannot follow this temporal pattern. The tight compaction of DNA and cessation of transcription during the final stages of spermatogenesis requires the synthesis of some mRNA transcripts before the corresponding proteins are needed. *Tnp1* and *Tnp2* are two such germ cell mRNAs that are stored in a silent state before translation is initiated. We identified several potential regulators of *Tnp* mRNA that are expressed at spermatogenic stages where translation is repressed.

The method we developed to isolate endogenous mRNAs and associated proteins from whole testis lysate provides the major benefit of assaying mRNA/protein complexes from an *in vivo* source containing all molecular components. Further, by targeting endogenous complexes, proteins are pre-bound and any mRNA modifications are accounted for. An important consideration is that we used testis lysate made from tissue containing a heterogeneous population of somatic and germ cells, including all stages of spermatogenesis. A benefit of the heterogeneity is that germ cells at all stages are probed, and in the case of *Tnp* mRNAs, we had the potential to identify candidate repressors and activators. The heterogeneity is also a drawback of the procedure because we do not know which cell type is the source of the specific mRNA. This is less important for *Tnp* mRNAs, since they are not expressed in somatic cells, but should be considered when applying the method to other target mRNAs. An alternative approach would be to isolate germ cells at specific stages, but this may not be a feasible option due to the loss of material and potential changes in mRNP complexes during the enrichment process. Another consideration when using this method is that we cannot distinguish between proteins that regulate disparate stages of the mRNA life cycle. We isolated proteins involved in splicing as well as translational regulators. Since RNA processing factors also can be translational regulators, we cannot differentiate between these roles.

Although we selected our candidate regulatory proteins from the testis proteome based on their association with *Tnp* transcripts, these five proteins possess known RNA-binding modules and RNA regulatory functions. CSDE1/Unr is a cytoplasmic protein with five cold-shock domains that plays many different roles in RNA processing and regulation, including regulating mRNA stability and translation through 3′UTR binding^28,29^. CSDE1 is necessary for mouse viability, and it has roles in mitosis, differentiation and apoptosis^30^. ELAVL1/HuR also plays a wide variety of regulatory roles in responding rapidly to extracellular signals, using its three RNA recognition motif (RRM) domains to target mRNAs with 3′UTR AU-rich elements and U-rich sequences and regulating their stability and translation^31^. Mice with conditional loss of ELAVL1 in germ cells exhibit male-specific infertility^32^. The mice have few spermatids due to cell death around meiosis, suggesting impairment of differentiation. These data are intriguing, given the beginning of *Tnp1* and *Tnp2* expression in round spermatids and our data showing high ELAVL1 expression in spermatocytes and round spermatids. IGF2BP3/IMP3 is expressed during embryonic development and is implicated in many cancers^33^. The IMP proteins contain two RRM and four K-homology (KH) domains, and IGF2BP3 localizes to mRNP granules, apparently sequestering mRNAs from degradation. The pentatricopeptide repeat (PPR) protein, LRPPRC/LRP130, interacts primarily with mitochondrial RNAs and controls RNA folding by binding to single-stranded regions^34^. However, a subset of LRPPRC is also in the nuclear fraction of a cell extract and localized at the nuclear and endoplasmic reticulum membranes^35,36^. MATR3 contains zinc finger and RRM domains, although it was identified first as a nuclear matrix component^37^. MATR3 controls RNA processing including alternative polyadenylation and intron inclusion^38^.

A growing body of work underscores the importance of RNA regulation at every step of spermatogenesis. Although there has been much discussion regarding *in vitro* spermatogenesis to allow reproduction by men with non-obstructive azoospermia (NOA), success will require deeper understanding of the biology of spermatogenesis from SSC to spermatozoa^39,40^. Further, for men with idiopathic infertility, identification of key molecules in spermatogenesis will allow testing, diagnosis, and ultimately treatment of these patients. Using a proteomics strategy, we identified proteins that associated with *Tnp* mRNAs, many of which have no known role in spermatogenesis and remain to be investigated. The five proteins we describe here are viable candidate regulatory proteins because they appear to be expressed in the cells where *Tnp* mRNAs are found. One of the associated proteins appears to be expressed at a low level, yet its detection by mass spectrometry suggests it was enriched. In the absence of additional data, LRPPRC may be considered a lower priority candidate. A *Tnp* regulatory protein must be expressed when the mRNA is present, but since most RNA regulatory proteins control many different RNAs, they are likely to be expressed in other cells as well. It is also entirely possible for RNA-binding proteins to be expressed and even associate with mRNAs, but only regulate their translation or stability when a full regulatory complex has formed. Further studies in animals will be required to conclude that candidate proteins affect TNP expression, since *Tnp* mRNA transcription and regulation occur only in specialized cell types during specific stages of spermatogenesis. Once *in vivo* effects are established, combinations of approaches, including using reporter assays or *ex vivo* systems, can be combined to elucidate detailed molecular mechanisms of how they may act on *Tnp1* and *Tnp2* mRNAs and how defects in their activities might contribute to human idiopathic sub- and infertility.

## Materials and Methods

### Animals

All animal procedures were approved by the Institutional Animal Care and Use Committee of the National Institute of Environmental Health Sciences (NIEHS) and were performed in accordance with the approved NIEHS animal study proposal (ASP 2014-0004).

### Testis protein isolation and western blots

Total protein lysate was prepared from the testes of DBA2/J mice (Jackson Labs) at 6, 10, 14, 20, 25, 30 and >150 days postpartum (dpp). The testes were minced with a clean scalpel then homogenized by vigorous pipetting in lysis buffer (150 mM sodium chloride, 0.5% w/v deoxycholate, 1% v/v Triton X-100, 0.5% w/v SDS, 50 mM Tris, pH 8.0) with 1X Halt™ Protease Inhibitor Cocktail (ThermoFisher). Protein concentration of lysates, diluted 1:100 or 1:1000, was determined via Bradford assay (Bio-Rad). For each gel lane, 30–40 μg of protein extract were mixed with NuPage LDS loading buffer containing 10% v/v beta-mercaptoethanol (BME, Invitrogen), boiled, and loaded onto 4–14% Bis-Tris NuPAGE gels (Invitrogen). Proteins were then transferred to nitrocellulose membranes (Invitrogen) for 45–60 min at constant 30 V. Membranes were immunoblotted with primary and secondary antibodies (Supplementary Table S1) for 90 and 45 min, respectively, with agitation at room temperature. Signal from bound antibodies was visualized using a LI-COR Odyssey imaging system.

### Oligonucleotide-mediated ribonucleoprotein complex purification

Neutravidin resin (Pierce) was centrifuged briefly (500 g × 1 min) and washed three times with PBS (prepared with DEPC-treated water). DNA oligonucleotides for RNA isolation with 3′ biotin were generated by Eurofins Genomics (Supplementary Table S2). Two nmol of biotinylated oligonucleotide was incubated with the resin while rotating at room temperature for 30–60 min and washed once with PBS. Whole testis lysate was prepared from adult mice by homogenizing tissue in NP40 lysis buffer containing Halt™ protease and phosphatase inhibitor (ThermoFisher) and RNase Inhibitor (Thermo Fisher). 5 mg of testis lysate was added to the biotinylated-oligonucleotide/resin mix and incubated while rotating at 4 °C for 1–2 h. After incubation, the volume was transferred into Micro Bio-Spin columns (BioRad) and centrifuged (500 g × 1 min). The column was washed three times with PBS. To elute target RNA, we added 3 nmol of competing non-biotinylated oligonucleotide, capped the column, and agitated at 60 °C for 10 min. Remaining protein was eluted by adding NuPage LDS loading buffer containing 10% v/v BME (Invitrogen) directly to the column, heating for 5–10 min at 95 °C, and then centrifuging (500 g × 1 min) to collect eluate. For protein analysis, the eluates were mixed with NuPage LDS loading buffer containing 10% v/v BME (Invitrogen), boiled and electrophoresed on 4–14% Bis-Tris NuPAGE gels (Invitrogen). Gels were stained with SimplyBlue SafeStain (Invitrogen).

### Mass spectrometric analysis

Proteins in gel slices were identified by mass spectrometry, as described previously^41^. Briefly, gel slices were excised and minced manually. Digests were then performed with a ProGest robotic digester (Genomic Solutions) where the gel pieces were destained by incubation in 25 mM ammonium bicarbonate/50% v/v acetonitrile (2 × 15 min). The gel pieces were dehydrated in acetonitrile, followed by drying under a nitrogen stream, and then incubated with 250 ng trypsin (Promega) for 8 h at 37 °C. The digests were collected and peptides were re-extracted three times. The extractions were pooled for each sample, lyophilized, and resuspended in 0.1% formic acid. NanoLC-ESI-MS/MS was performed using an Agilent 1100 nanoLC system on-line with an Agilent XCT Ultra ion trap mass spectrometer with the chip cube interface. The peptide digest was loaded onto an Agilent C18 chip (75 μm × 43 mm) followed by a 15-min wash with 5% v/v acetonitrile/0.1% v/v formic acid. Peptides were eluted with a gradient of acetonitrile as follows: 0–45 min, 5–50% v/v acetonitrile; 45–50 min, 50–95% v/v acetonitrile; 50–60 min, acetonitrile maintained at 95%. The mass spectrometer was used in the positive ion, standard enhanced mode and included settings of a mass range from 200 to 2200 m/z, an ionization potential of 2.1 kV, an ion charge control smart target (number of ions in the trap prior to scan out) of 100,000 or 200 ms of accumulation, and a 1.0-V fragmentation amplitude. MS/MS data were acquired using a data-dependent acquisition format with the six most abundant ions from each MS scan further interrogated by MS/MS. The automated switching for MS/MS required a threshold of 5000 counts.

The Spectrum Mill software suite from Agilent was used to generate peaks lists from the data obtained from each NanoLC-ESI-MS/MS analysis using the data extractor feature. The data extractor settings included filtering for deconvoluted ions observed between 300 and 5000 Da and a retention time between 10 min and 60 min. MS scans with the same precursor mass (±1.5 m/z) and retention time within 30 s were merged. Moreover, of the remaining MS/MS spectra, only spectra that contained sequence tag information greater than two residues were submitted for database searching. The resulting extracted data were then searched against the SwissProt/UniProt database (release 2016_01) using the MS/MS search function in the Spectrum Mill software. Search settings included a trypsin specificity with up to two missed cleavages allowed, a precursor ion mass tolerance of 1.5 Da, a product ion mass tolerance of 1.0 Da, variable methionine oxidation, and a minimum matched spectral intensity of 60%. To analyze candidate regulatory proteins, we calculated normalized spectral counts for proteins associated with *Tnp1* or *Tnp2* by dividing the *Tnp* spectral counts by the *Actin* spectral counts (Supplementary Dataset S1). If the value for *Actin* was zero, we divided by 0.9, a conservative value to prevent overestimating the relative abundance.

### mRNA isolation and RT-qPCR

RNA from testes and sucrose gradient fractions was isolated using Trizol per manufacturer’s instructions (Life Technologies). RNA from all experiments was stored at −80 °C if not used immediately. RNA was treated with DNase I (New England Biolabs) for 10 min at 37 °C, and then the enzyme was deactivated for 10 min at 75 °C. Quality and concentration of RNA were assessed by absorbance at 260 and 280 nm using a NanoDrop 2000c spectrophotometer (ThermoFisher). 500 ng of each RNA was used as template for cDNA synthesis with iScript Reverse Transcription Supermix (Bio-Rad), which uses a blend of oligo(dT) and random hexamer primers. For each 20 μl cDNA synthesis reaction, 1 μl of reverse transcriptase was used, except in negative control reactions where water was substituted for enzyme. The reaction protocol was carried out as directed by manufacturer: 5 min at 25 °C, 20 min at 46 °C, and 1 min at 95 °C. All cDNAs were stored at −20 °C if not used immediately. Custom RT-qPCR primer sets (Integrated DNA Technologies) span introns where possible, and the sequences are in Supplementary Table S3. Primer sets were tested with samples from control cDNA synthesis reactions lacking reverse transcriptase to detect genomic contamination, and all primer sets failed to amplify from these samples, as defined by a C_q_ < 38. Primer sets were also assessed by BLAST against the mouse genome and transcriptome to ensure specificity. Specificity of primers was validated by melt curve analysis, with each primer set containing a single peak ranging from 83.5–85 °C. Standard curves were determined over a 3–4 log dynamic range and allowed for determination of R^2^, slope and PCR efficiency (see Supplementary Table S3). RT-qPCR was performed using a Bio-Rad CFX-384 or CFX-96 Real-Time PCR System in Hard-Shell PCR Plates (Bio-Rad) with the program CFX Manager 3.1 (Bio-Rad). Each 10 μl RT-qPCR reaction contained 5 μl SsoAdvanced^TM^ Universal SYBR Green Supermix (Bio-Rad), 10–50 ng cDNA, and 250 nM forward and reverse primers. The thermocycling parameters began with incubation at 95 °C for 30 s, followed by 40 cycles of 95 °C for 5 s and 60 °C for 15 s. Mouse *Actin* RNA was used as a reference transcript in our study using previously validated primer sets^42,43^. Efficiencies of mRNA isolation were calculated by normalizing to *Actin* input mRNA measurements and then calculating the transcript abundance relative to the level of the target mRNA in the input extract using the ∆∆C_t_ method. The specificities of mRNA isolation were calculated by normalizing to *Actin* input mRNA measurements and calculating the transcript abundance relative to the level of the selected mRNA using the ∆∆C_t_ method. Three technical replicates were performed for each biological replicate.

### Sucrose density gradient centrifugation

On the day of the experiment, testes were excised from mice, and the tunica was removed and washed once in cold PBS. For each sample, one testis was placed in 1 ml of complete lysis buffer (20 mM HEPES/KOH pH 7.4, 0.1 M KCl, 5 mM MgCl_2_, 0.2 mg/ml cycloheximide, 1X Halt^TM^ Protease Inhibitor cocktail [ThermoFisher], 40 U/ml RNaseOUT^TM^ [Invitrogen], 1.5% w/v dodecylmaltoside). Tubules were mechanically disrupted and homogenized in lysis buffer with gentle teasing apart and pipetting. Cell debris was removed by centrifugation at 12,000 g for 20 min at 4 °C. Supernatant was layered onto a 15–50% w/v sucrose gradient prepared in buffer containing 200 mM KCl, 20 mM HEPES/KOH pH 7.4, 15 mM MgCl_2_, 1 mM DTT, 0.2 mM cycloheximide, 40 U/ml RNaseOUT^TM^ and DEPC-treated H_2_O. Gradients were centrifuged at 35,000 rpm for 3 h at 4 °C in a Beckman SW41 rotor. Gradients were displaced from the bottom with a 60% w/v sucrose solution, and a continuous 254 nm absorbance profile was recorded; the sample was collected into 12 fractions and stored on ice for immediate RNA isolation. Absorbance readings were collected via TRACRDAQ software and analyzed using Graphpad Prism7.

### Immunohistochemistry

Testis sections were prepared from adult DBA2 mice by fixation in 10% v/v neutral-buffered formalin for ~24 h followed by paraffin embedding and sectioning at 5 μM thickness. At the time of staining, slides were deparaffinized by two 15-min incubations with xylenes (Sigma), followed by serial rehydration with an ethanol series (100%, 95%, 80%, 70%, 50%, 25% v/v), each incubation lasting 10 min. Antigen retrieval was performed for 20 min by incubation at 95 °C in sodium citrate buffer (10 mM sodium citrate, pH 6; 0.05% v/v Tween-20). Slides were blocked with a buffer containing 5% v/v serum from the secondary antibody host species, 3% w/v BSA, and 0.1% v/v Triton X-100 for 30 min at room temperature, stained with primary and secondary antibodies (Supplementary Table S4), and fixed with ProLong Gold with DAPI (Invitrogen). Lectin peanut agglutinin (PNA) staining was performed experimentally as a secondary antibody. Visualization and image capture were performed on a Zeiss AxioObserver Z1 fluorescence microscope (Carl Zeiss Inc, Oberkochen, Germany) using a Plan-Apochromat 20 × /0.8 DIC objective with a metal halide light source. The DAPI nuclear stain was visualized with Zeiss filterset 49 (335–383 nm excitation, 420–470 nm emission) with a Zeiss AxioCam MR R3 camera. Subsequently, Zeiss filterset 38 (450–490 nm excitation, 500–550 nm emission) was used for excitation of Alexa488. Zeiss filterset 43 (538–562 nm excitation, 570–640 nm emission) was used to collected images of Alexa568 signal. Finally, Zeiss filterset 50 (625–655 nm excitation, 665–715 nm emission) was used to collect images of Alexa647 signal.

## Supplementary information

Supplementary Figures 1-5

Dataset 1

## Data Availability

The unfiltered mass spectrometry dataset generated and analysed for this study is available as Supplementary Dataset S1. Mass spectrometry data have been deposited to the ProteomeXchange Consortium via the PRIDE^43^ partner repository with the dataset identifier PXD015384.
