# Supplementary material for: Mass spectrometric identification of candidate RNA-binding proteins associated with Transition Nuclear Protein mRNA in the mouse testis
Source: Sci Rep. 2019 Sep 20;9:13618. doi: 10.1038/s41598-019-50052-z (PMC6754440; doi:10.1038/s41598-019-50052-z)
Supplement: Supplementary file 1 — Supplementary Figures 1-5 [file 41598_2019_50052_MOESM1_ESM.pdf]

Supplementary Information:

Mass spectrometric identification of candidate RNA-binding proteins associated with *Transition Nuclear Protein* mRNA in the mouse testis

Bart T. Phillips, Jason G. Williams, Dustin T. Atchley, Xiaojiang Xu, Jian-Liang Li, Andrea L. Adams, Katina L. Johnson, and Traci M. Tanaka Hall*

Epigenetics and Stem Cell Biology Laboratory, National Institute of Environmental Health Sciences, National Institutes of Health, Research Triangle Park, NC 27709, USA

*Correspondence: hall4@niehs.nih.gov

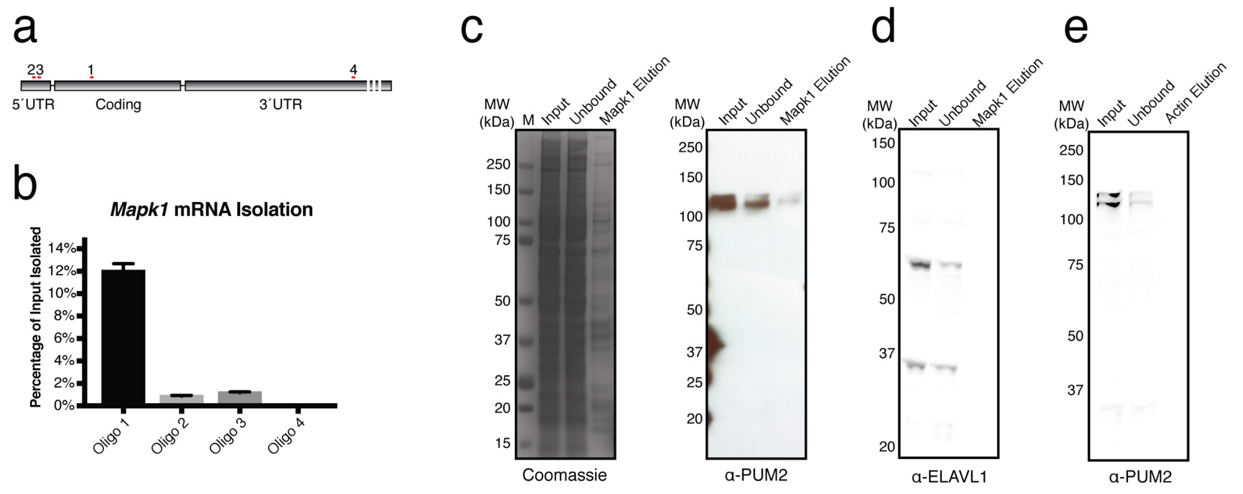

***Mapk1* mRNA is efficiently isolated with known binding partner PUM2**. (**a**) Schematic representation of *Mapk1* mRNA. Four oligonucleotides targeting unique sequences and regions of the mRNA are numbered and shown. (**b**) A bait oligonucleotide complementary to the *Mapk1* coding sequence most efficiently isolated the target transcript. The amount of endogenous *Mapk1* mRNA selected by each bait oligonucleotide was assessed by RT-qPCR and is reported here as a percentage of mRNA in the input extract. The error bars represent the SEM for three biological replicates. Oligonucleotide 4 did not capture a detectable amount of *Mapk1* mRNA (C_t>38). (**c**) PUM2 co-purifies with *Mapk1* mRNA. Coomassie-stained gel (left) of input testis extract, unbound fraction, and *Mapk1* oligonucleotide elution sample and a corresponding anti-PUM2 western blot (right) are shown. PUM2 western blots and immunoprecipitations consistently reveal a doublet for this and other PUM2 antibodies[41]. (**d**) The abundant RNA-binding protein, ELAVL1, does not co-purify with *Mapk1* mRNA. An anti-ELAVL1 western blot of input testis extract, unbound fraction, and *Mapk1* oligonucleotide elution sample is shown. ELAVL1 is a 36 kDa protein, corresponding to the faster migrating band. The slower migrating band at ~60 kDa may be a crossreactant or a persistent dimer of ELAVL1. Both bands are absent from the oligonucleotide eluted sample. (**e**) PUM2 does not co-purify with negative control *Actin* mRNA. An anti-PUM2 western blot of input testis extract, unbound fraction, and *Actin* oligonucleotide elution sample is shown.

Suppl. Figure S2

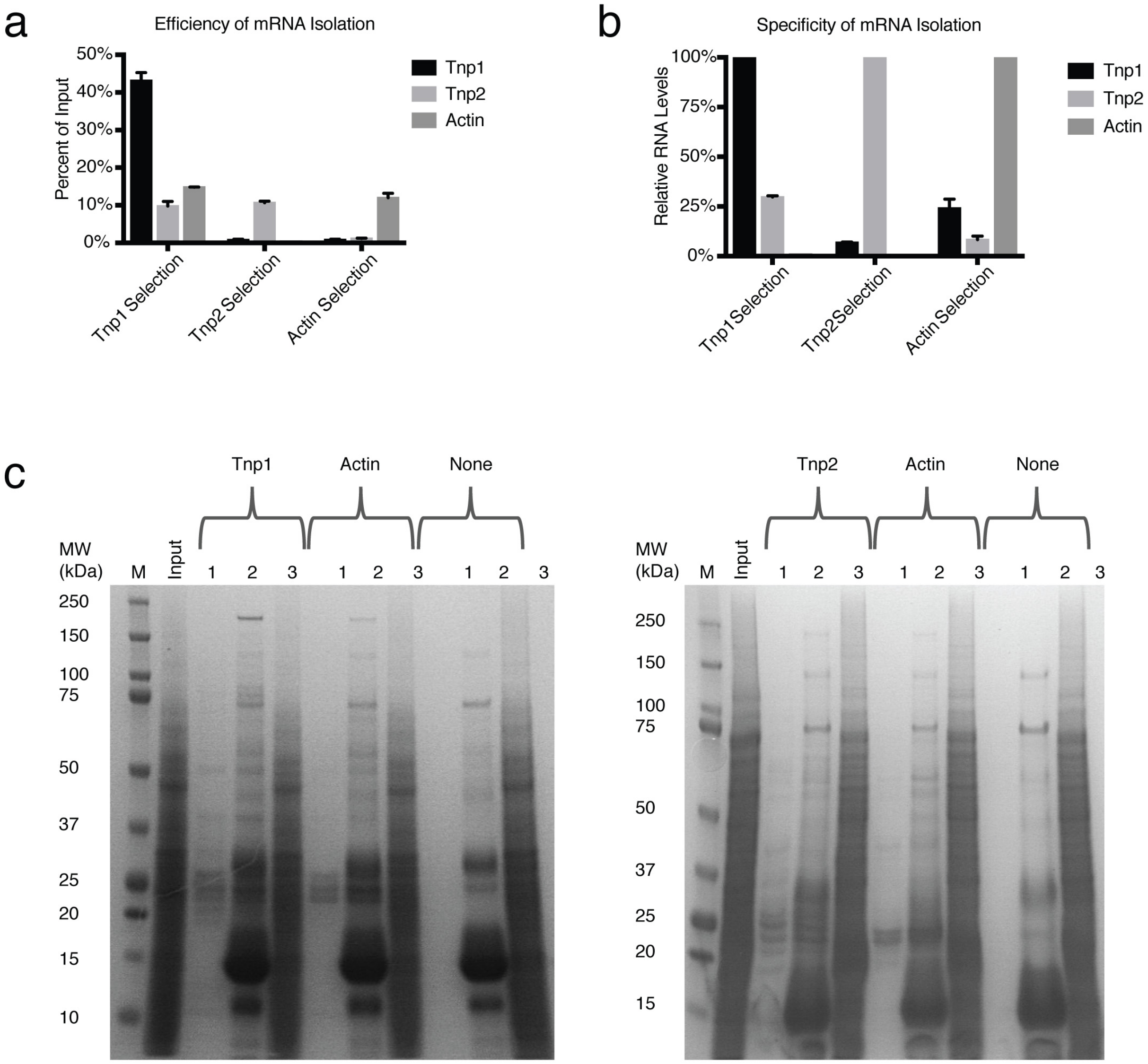

Efficiency and Specificity of *Tnp1* and *Tnp2* mRNA Selection. (**a**) Efficiency of target mRNA selection from mouse testis lysate using biotinylated oligonucleotides complementary to their coding regions. The percentage of input transcript selected in each mRNA isolation experiment was determined by RT-qPCR. Error bars represent the SEM of three technical replicates. In each case, the selected mRNA was more efficiently selected than the non-selected *Tnp* or *Actin* mRNAs. (**b**) Target mRNAs are preferentially selected from mouse testis lysate. The amounts of 'off-target' transcripts isolated were determined by RT-qPCR and are shown as percentages relative to the specific mRNA targeted with the bait oligonucleotide. Error bars represent the SEM of three technical replicates. (**c**) *Tnp1* and *Tnp2* mRNAs co-purify with populations of proteins. Representative Coomassie-stained gels of elution samples from *Tnp1* (left) and *Tnp2* (right) mRNA-mediated purification. Input lysate shows total protein before isolation. Controls were performed with *Actin* mRNA selection or with no bait oligonucleotide (None). For each isolation, lane 1 is the *Tnp1* or *Tnp2* oligonucleotide elution, lane 2 is proteins that remained after oligonucleotide elution and removed by boiling the resin in gel loading buffer, and lane 3 is the unbound fraction. M contains molecular weight marker standards.

Suppl. Figure S3

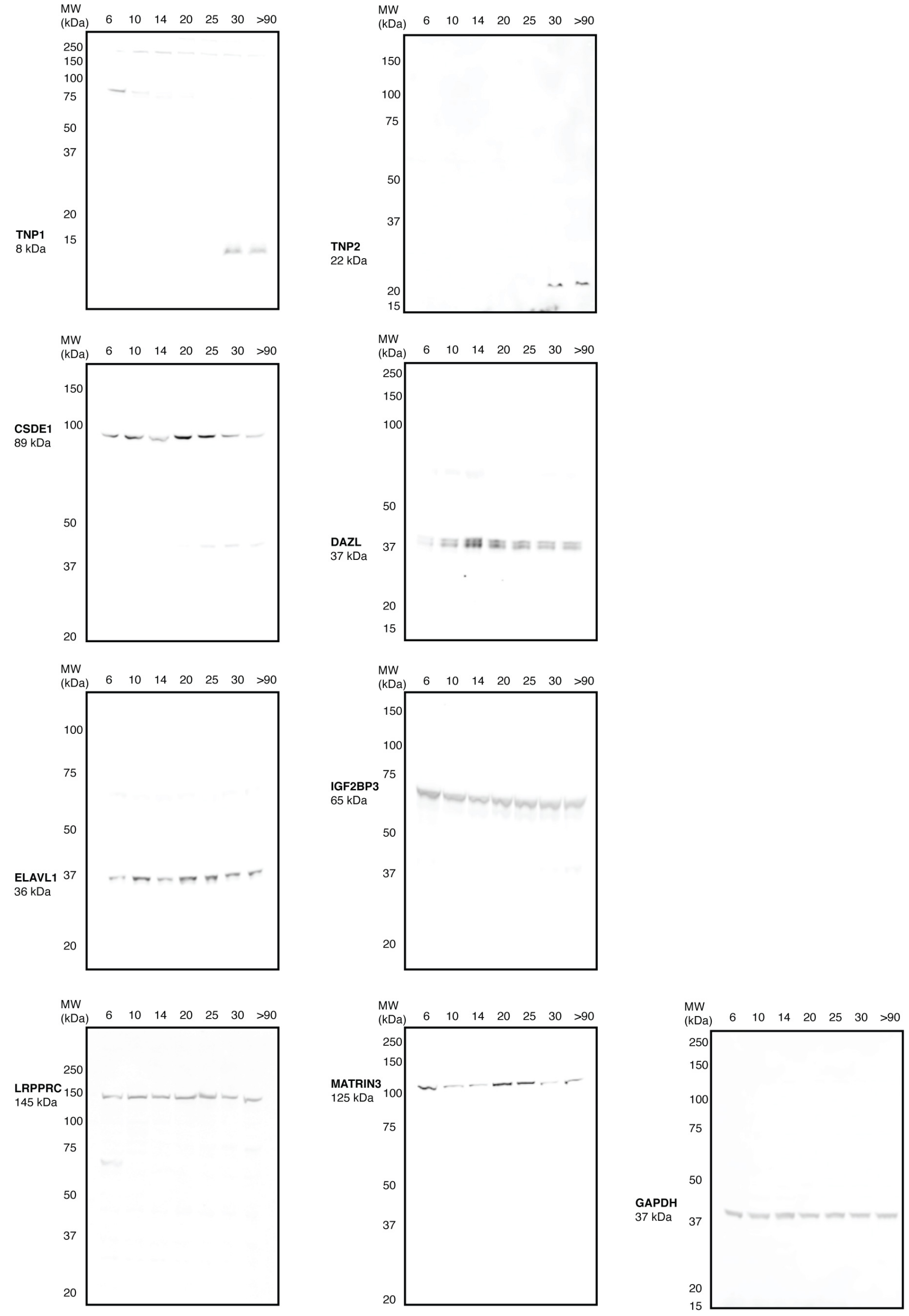

Specificities of anti-TNP1, TNP2, and candidate regulatory protein antibodies. Representative Western blots from Fig 2B are shown in their entirety. Three biological replicates were performed for each experiment. Extracts were prepared from mouse testes at seven distinct developmental time points (6, 10, 14, 20, 25, 30 and >90 dpp).

Suppl. Figure S4

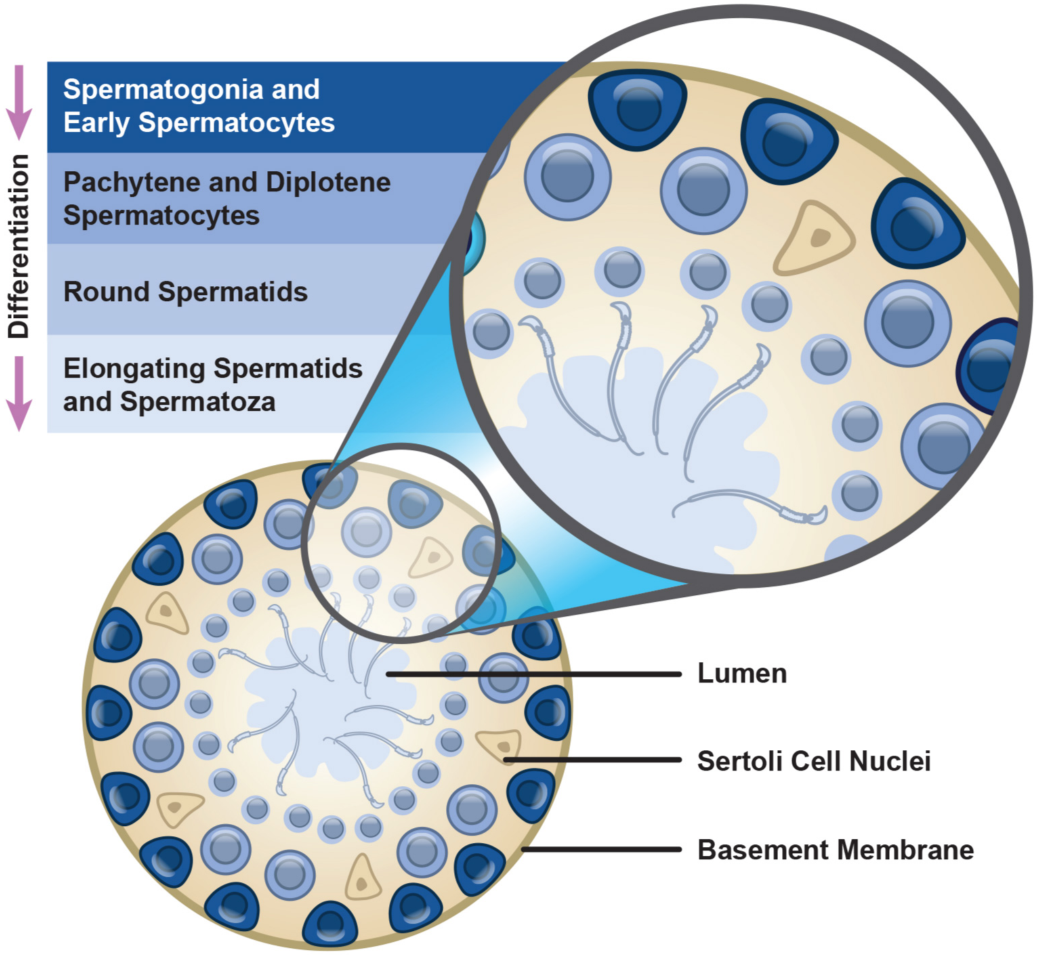

A mouse seminiferous tubule section comprises associated germ cells at different steps of differentiation with discrete locations and morphologies. Spermatogenesis proceeds through defined steps as germ cells differentiate and mature. Because the timing of maturation steps is consistent, a particular cross section of the mouse seminiferous tubule will contain multiple types of germ cells at distinct steps of differentiation with the less differentiated forms closer to the basement membrane and the most mature near the lumen. Image courtesy of NIEHS.

Suppl. Figure S5

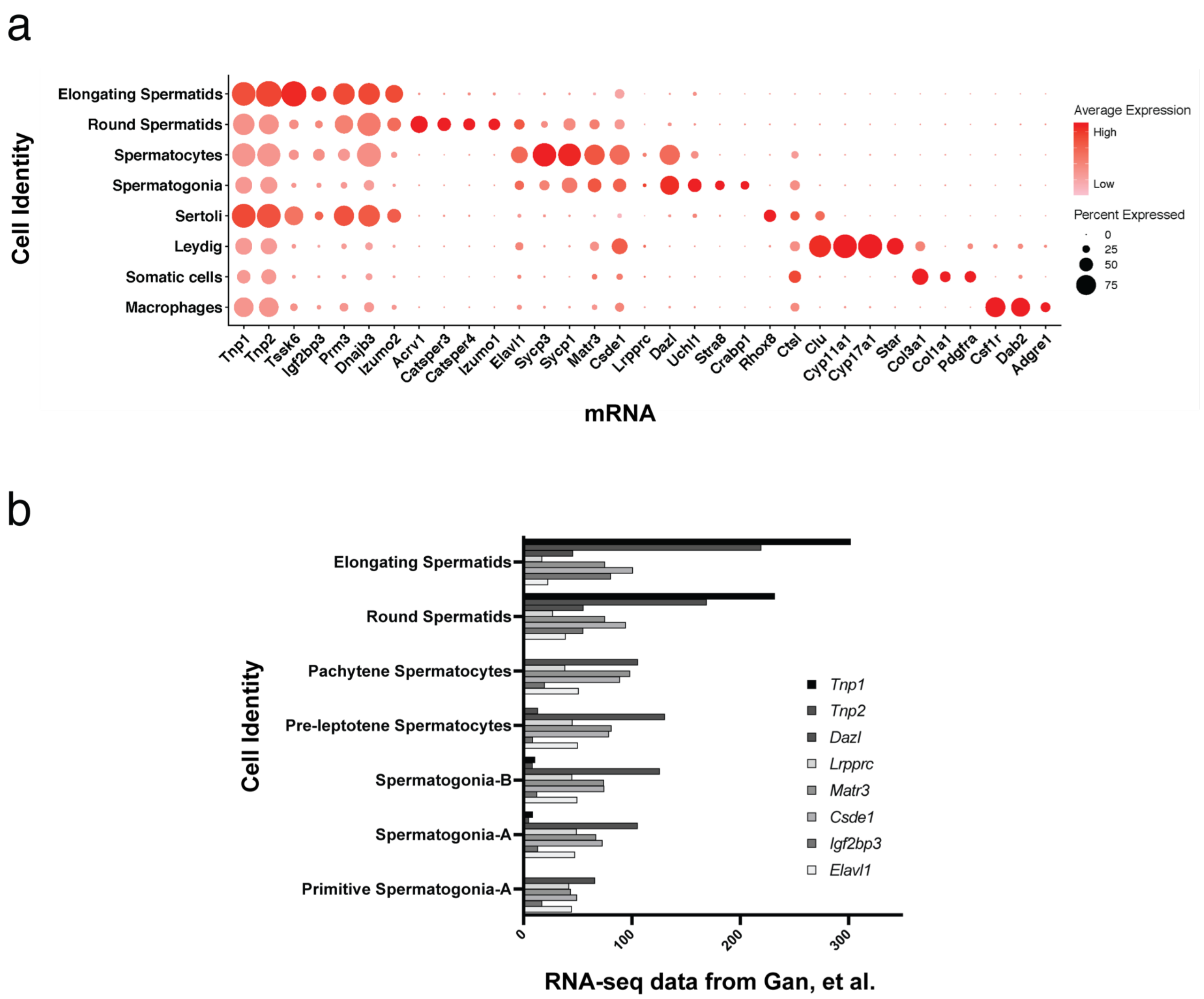

Expression of candidate genes in testis cell types in published RNA-seq data sets. (**a**) A dot plot illustrating mRNA expression of candidate regulators and known cell type markers for each major cell type in the adult mouse testis. The mouse single cell RNA-seq raw data were downloaded from the GEO (GSE121904: PND30, Ad.ACRV1-D and Ad.THY-E samples), pre-processed by Cell Ranger v 3.0.1, corrected for batch effect using Harmony, and annotated for major cell types with Seurat v 3.0.2. The marker genes for known cell types were based on those reported by Grive, et al. 2019[17]. The size of the individual dots reflects the proportion of cells for each cell type expressing the candidate regulators or marker genes and the color reflects the relative expression level of each gene across all cells of a particular type. (**b**) Bar graph illustrating mRNA expression of candidate regulators in bulk RNA-seq data from isolated spermatogenic cell types. The data for candidate gene expression in the different cell types were selected from Supplementary Data 2 reported by Gan, et al. 2013[18].
